# Scaling up an intervention to protect preterm infants from neurodevelopmental disabilities — findings from a qualitative process evaluation comparing standard with enhanced quality improvement support packages for maternity units in England

**DOI:** 10.1186/s13012-023-01275-2

**Published:** 2023-05-24

**Authors:** Sabi Redwood, Christalla Pithara-McKeown, Tracey Stone, Emma Treloar, Jenny L. Donovan, Karen Luyt

**Affiliations:** 1 Population Health Sciences, The National Institute for Health and Care Research Applied Research Collaboration West (NIHR ARC West) at University Hospitals Bristol and Weston NHS Foundation Trust, Bristol Medical School, University of Bristol, Bristol, UK; 2 Population Health Sciences, Bristol Medical School, University of Bristol, Bristol, UK; 3 University Hospitals Bristol and Weston NHS Foundation Trust, Bristol, UK

**Keywords:** Quality improvement, Normalisation process theory, Normative restructuring, Relational restructuring, Sustainment

## Abstract

**Background:**

A quality improvement strategy (PReCePT) was used in a standard and enhanced format to scale up a clinical intervention (administering magnesium sulphate to women in preterm labour) across all maternity units in England to protect prematurely born infants from neurodevelopmental disabilities. Formal evaluations reported the effectiveness of the standard package alone in increasing the administration of magnesium sulphate. In this paper, we focus on the findings of the process evaluations, using normalisation process theory to explain how different implementation contexts generated the observed outcomes relating to normative and relational restructuring and sustainment.

**Methods:**

Interviews were conducted with key individuals in implementation of leadership positions nationally and locally. Interviews were analysed initially using the framework method. We then engaged recursively with NPT constructs to generate generalisable insights with pragmatic applicability in other settings.

**Results:**

In total, 72 interviews were conducted with good representation from units across England and staff from the National Academic Health Science Network. We found that all units irrespective of whether they received a standard or enhanced QI package were successful in the ‘normative restructuring’ of their setting to enable magnesium sulphate to be administered. This suggests that this implementation outcome is necessary to achieve improvements. However, it may not be sufficient to sustain the changes once additional resources have been withdrawn. Sustainment, our findings suggest, required ‘relational restructuring’ to accommodate altered workflows and facilitate the sharing of responsibilities and tasks in daily practice. Relational restructuring was more likely to have been achieved units receiving enhanced QI support but also happened in units with standard QI support, especially in those where perinatal team working was already well established.

**Conclusion:**

Unlike other large QI-focused spread-and-scale programmes which failed to show any impact on outcomes, the PReCePT programme in both the enhanced and standard support packages led to improvements in the uptake of magnesium sulphate. The findings suggest that QI programmes interact with the enabling factors, such as strong interprofessional team working, already present in the setting. A standard package with minimal support was therefore sufficient in settings with enabling factors, but enhanced support was required in units where these were absent.

Contributions to the literature

- Normative restructuring, or changes that are produced in the norms, rules and processes as a result of implementing a new intervention, was necessary to make clinical practice improvements. However, it was not sufficient to achieve sustainment.
- Relational restructuring, or changes in the way people organised and related to each other, was found to be crucial in maintaining improvements in clinical practice. This was more likely to occur in units with already established perinatal team working and in those receiving enhanced support to promote changes in behavioural norms.

## Background

Learning from different approaches enabling the uptake of evidence-based interventions across organisations and health systems is crucial so that more people can benefit from innovations in healthcare [1]. Yet spreading and scaling an intervention, even when it is evidence based and consistent with clinical practice guidelines, are complex and dependent on implementation contexts [2–4]. The term ‘spread’ refers to replicating a change based on an innovation or new intervention in a different setting from that in which it was originally developed. Scaling up, which is part of spread, means the use of a guided process enabling the new practice to be adopted, usually through involvement of a ‘higher-level’ entity such as a professional body or government agency [1, 2]. Despite efforts to generate systematic methodologies for implementation, spread and scale-up of interventions and evidence-based clinical practice guidelines to optimise care across health systems [5], there are no universally replicable methods, because of the complex networks of interactions and articulated tasks in healthcare delivery [4]. Instead, learning and insights into how successful changes were accomplished and sustained will be developed through an accumulation of fine-grained accounts describing what was done to implement, spread and scale up an innovation, what changed as a result and, crucially, why and how it changed. This allows comparisons to be made between the contexts, mechanisms and outcomes of implementation processes which change over time and between different settings [6] and how processes and their contexts shape each other in the complex adaptive systems of healthcare delivery [7].

In this paper, we describe how a perinatal quality improvement (QI) strategy was used in a standard and an enhanced format to scale up a clinical intervention to protect prematurely born infants from cerebral palsy and other neurodevelopmental disabilities which cannot be cured. Since 2015, the UK governmental body of the National Institute for Health and Care Excellence has recommended that women presenting in preterm labour are given an intravenous infusion of an inexpensive drug, magnesium sulphate [8]. Yet despite the evidence that magnesium sulphate (MgSO4) significantly reduces the risk of disabilities to premature infants under 30 weeks’ gestation, by 2017, only 64% of eligible women were receiving it, while high regional variation in uptake suggested serious inequalities in perinatal care [9].

In order to raise awareness and increase MgSO4 administration to all eligible women during preterm labour, the West of England Academic Health Science Network (AHSN) in collaboration with University Hospitals Bristol and Weston NHS Foundation Trust used QI methodology and coproduction principles to develop a perinatal QI intervention, the Prevention of Cerebral Palsy in PreTerm Labour (PReCePT) programme [10]. PReCePT was piloted and refined in five maternity units before being selected for adoption and spread across the national AHSN Network during 2018–2020. The aim of the National PReCePT Programme (NPP) was to support maternity units to increase their average uptake of MgSO4 to eligible mothers to 85% by 2020. A randomised controlled trial (the PReCePT QI study) was embedded in the NPP to assess whether a standard QI support package (used in the NPP) was sufficient for a national scale-up of the QI intervention or whether a more resource-intensive, enhanced support package was needed for successful scale-up. For a comparison between the standard and enhanced QI packages, see Table 1. The components of the packages were intended to be used flexibly by implementers according to local needs.

**Table 1:** Comparison between the components of the standard and enhanced QI packages

	**Standard support**	**Enhanced support**
**PReCePT QI toolkit**	•Clinical guidance •Preterm labour proforma template •Staff training presentations •Parent leaflet •Posters for display on the unit to raise staff awareness •QI learning log •Project dashboard •Pens, magnets, lanyards and other aide-mémoires to promote MgSO4 to unit staff (available for purchase only)	As per standard support
**Implementation guidance**	PReCePT implementation guide	PReCePT implementation guide *PLUS* PReCePT ‘how-to’ guides, with more specific instructions developed through the pilot phase
**QI training session**	Regional level QI training and guidance to adapt materials for local use, cascaded from AHSN	As per standard support
**Regional support**	Support from a regional level neonatal lead and AHSN lead	As per standard support
**Local obstetrician champion**	Local obstetrician identified by unit to guide and oversee local implementation	As per standard support (named as joint PI, at discretion of local site)
**Funded time for local midwife champion**	Funded time of up to 90 h per unit (on average 2 h per week)	As per standard support, *PLUS* funding for up to 90 extra hours backfill, on average over 12 months
**Funded time for local neonatologist champion**	None	Funded time for a local neonatologist PI, working on average 0.5 PA (2 h) per week over 12 months, to provide clinical leadership in local unit (fixed-term contract or secondment from an NHS organisation) (N.B. 0.5 PA backfill could be split with obstetrician PI, at discretion of local site)
**QI coaching**	None	Structured coaching in local unit from an experienced QI coach. To include the following: •First visit where the QI coach worked with local unit to create a bespoke implementation plan •Telephone coaching in liaison with the local champion(s), with occasional face-to-face visits as logistics permit •Ongoing dedicated support to help embed the QI toolkit within local unit •Final visit to support local unit to tie up data collection and plan for ongoing sustainability
**Learning events**	None	Funding for up to three members of staff from local unit to attend three learning events. These bespoke learning events were held every 2–3 months during the period of implementation and brought together teams from other enhanced support units to share their activity and learning on how they are implementing the PReCePT QI toolkit and working to address issues and challenges
**Collateral funding**	None	Provision of a tablet computer to be used by the local midwife champion to micro-coach colleagues, plus a small fund for purchasing study collateral (pens, magnets, lanyards, aide-mémoires), if required
**Celebration event**	None	Funding for up to three members of staff from local units to attend a celebration event which brought together teams from all enhanced support units to wrap up the study and to share experiences, learning and successes

Evaluations of the NPP, and the PReCePT study, have reported the effectiveness of the standard package alone in increasing the uptake of MgSO4 across all units in England [11, 12]. The qualitative process evaluation of the PReCePT QI study also suggested that the standard package alone may be adequate for units delivering care within an implementation-enabling environment, but a more intensive support package may be needed to enable meaningful and sustained change in maternity units with less enabling environments [11]. In this paper, we focus on the findings of the process evaluations of the NPP and PReCePT QI study, using normalisation process theory (NPT) [6, 13]. NPT is a theory of implementation, developed to assist in identifying the components and elements of implementation processes being empirically studied. We selected it for this study to focus attention on individual and collective behaviours necessary to incorporate and scale up a new activity — in this case administering MgSO4 to eligible women in preterm labour — into routine clinical practice across a health system.

We used NPT to conceptualise how the strategic intention of the AHSN network (to increase the number of eligible women who actually receive MgSO4, thus protecting their premature infants from neurodevelopmental disorders) is translated into the everyday practices of others (staff working in all English maternity units). The national AHSN network and the regional AHSNs acted as the ‘higher-level entity’ [1] or support-system level organisation [14] which coordinated and supported local implementation. The translational activities were rooted in QI methodology but delivered in two different ways: the standard package that was supported by the regional AHSNs and the enhanced package which included, in addition to the SSP, intensive engagement with QI coaches, backfill funds for local clinical champions on top of those offered in the SSP and national networking and celebration events. These support activities affected and shaped the implementation context, defined in NPT as the patterns of social relations and structures that unfold over time, make up the implementation environment and promote or inhibit the mobilisation of resources for implementation [7]. We sought to bring to the surface some of the mechanisms that motivated and shaped implementation processes, how they were used to achieve the strategic intention and how individual and collective action led to the implementation outcomes. The outcomes refer to the effects of the mechanisms at work in producing changes in practice and social relationships, and how these became embedded in routine clinical practice, in this case to achieve a MgSO4 administration rate of 85%.

## Methods

The overall design of the mixed-methods evaluation of the PReCePT programme has been described elsewhere [11, 12]. We conducted a process evaluation to describe how the standard and enhanced QI packages were implemented and to explain differences or similarities.

### Setting

One-hundred and fifty maternity^1^ units in England were enrolled in the NPP, funded by NHS England, and roll-out was overseen and managed by the regional AHSNs who were responsible for providing implementation support to individual units. Funding was allocated to AHSNs for the recruitment of regional clinical leads (either obstetricians or neonatologists) who provided clinical oversight and support to units. Embedded in the NPP was the PReCEPT study, which randomised 40 units to the standard support package (SSP), i.e. aligned with the NPP, or to the enhanced support package (ESP). ESP teams had access to NPP support resources and AHSN support but in addition received intensive QI coaching tailored to individual units’ needs and implementation readiness.

### Data collection

Interviews were conducted with key individuals in leadership positions in the AHSNs (those providing QI and implementation-related leadership and regional clinical leads providing clinical leadership to units in their region) and in the maternity units (midwifery, neonatal and obstetric ‘champions’ or leads who provided clinical leadership for implementation). Our recruitment strategy was guided by the principles of information power [15] so that sample size was driven by the relevance and extent of the knowledge and experience held by participants rather than predetermined numbers. Interview guides for both groups were developed in collaboration with the project steering groups to ensure all relevant aspects of implementation were addressed. A question relating to the role of COVID-19 pandemic in the uptake of MgSO4 was added in 2020.

### Data analysis

The process evaluation data were initially analysed inductively using the framework method [16]. This method facilitates the production of highly structured outputs of summarised data in a matrix or framework. In this way, data were compared and contrasted across as well as within cases to identify and describe factors affecting implementation and observed outcomes. Cases in this study referred to maternity units. We then engaged in an iterative process using abduction [17, 18] to look for explanations in our empirical data related to NPT and to draw out inferences orientated towards developing the theory^2^ [13]. We paid close attention to how the two support packages were used to translate the strategic intention of increasing the administration of MgSO4 into practical changes in the way work was organised in everyday practice [13].

The aim was to capture how different implementation contexts and perinatal team^3^ dynamics — frequently lost in accounts of barriers and facilitators [19] — generated the observed implementation outcomes. In this paper, we focus on the implementation outcomes, defined as the effects that make visible how things change as implementation processes proceed [13] to enable a more nuanced understanding of how people have to adapt their practice and how implementation resources can support these adaptations. The outcomes we focus on are normative restructuring, relational restructuring and sustainment. We used these NPT constructs to help explain some of the differences and similarities observed between the units receiving SSP and ESP.

## Results

In total, 72 interviews were conducted with good representation from units across England and staff from the national AHSN network. Eighteen interviews were conducted with staff from units receiving ESP, comprising nine midwives, four obstetricians and five neonatologists; 33 interviews were conducted with staff from units receiving SSP, comprising 13 midwives, 10 obstetricians and 10 neonatologists. In addition, we interviewed nine regional AHSN leads responsible for the roll out of the NPP and 12 AHSN staff who worked with clinicians in local maternity units.

The clinical intervention to be scaled up involved the administration of an intravenous loading bolus dose of MgSO4, a relatively inexpensive drug, followed by a maintenance infusion.

Although relatively straightforward, what the initial analysis suggested is that the intervention destabilised the conventional professional organisation, or normative structure, of care for women in labour and preterm infants that resulted from introducing the treatment. The care of women was the responsibility of obstetricians and midwives, whereas the care of the infant was that of neonatologists and neonatal nurses. Preventing neurological damage to the preterm infant meant that MgSO4 had to be administered to women before birth — the responsibility of obstetric teams for the benefit of preterm infants who are the responsibility of neonatal teams. This structuring of responsibilities had practical implications because maternity and neonatal units were not always colocated at hospital sites, and this physical distance led to fewer opportunities for communication among staff. Other structural barriers included regulations about who can prescribe and administer medications and access to medical notes which are separate for mother and baby and held in separate physical locations or databases. ‘Ownership’ of PReCePT was therefore initially contested, but it was a crucial aspect of its success. The following interview excerpts illustrate the discussions around which professional group should provide leadership for implementing the required changes in clinical practice:

[Before the PReCePT intervention] it [was] the neonatologists trying to tell the obstetricians what to do and how to look after their patients. […] And that was quite frustrating that people weren’t implementing it and then when PReCePT came in, they suddenly were. And nothing new really, there wasn’t new data that came on board, it was just someone different telling them [obstetricians] should do it. (P26, Neonatologist, ESP Unit31)

It makes sense that actually this is an obstetric project really rather than sit with neonatology which was the original thinking […] It sits better with maternity because [they] are the ones who have to administer it […] with an obstetrician who has actually been doing degrees in pre-term deliveries so it made sense for that person [to lead]. (P46, Neonatologist, SSP Unit12)

What this shift in responsibility for leading the change signals is that it is not the technical complexity of the clinical intervention that needed to be addressed in implementation and spread, although it did require additional steps in workflows and time, mainly for midwives. Rather, it was the work of integrating the change into the wider ecology of clinical practice including the policy/regulatory context and the organisational, team and individual practitioner levels that required considerable thought and effort [20]. In the following sections, we focus on three of the NPT constructs relating to outcomes that explain how this work was accomplished: normative restructuring, relational restructuring and sustainment [13].

### Normative restructuring

Normative restructuring refers to the changes that were required to increase MgSO4 uptake: changes to the norms, rules and resources that govern the actions of maternity and neonatal teams. An important precondition for starting the work was an acknowledgement that the change in clinical practice was necessary. Some clinicians assumed that the administration of MgSO4 was already routine and were surprised to learn that following the collection of baseline data for all maternity units, the actual recorded rates of administration did not align with their expectation, highlighting the value of real-time and accurate data collection, as suggested below:

Everyone’s first response is, ‘we already give it and it is a normal part of our everyday care when we are caring for women in prem labour’. But actually the data didn’t support that. (…) One of the first things we did when the project came in [was to feed back their administration data] because everybody was so adamant that they did do it already. (NPP support, AHSN6)

The restructuring activities observed in all SSP and ESP units included modifications to the professional organisation of care for women in labour and preterm infants, to clinical guidelines and processes and to procedures for documentation and communication across the perinatal team and with other members of the multidisciplinary team involved in care of women in preterm labour. These included anaesthetists and pharmacists, and settings outside the labour ward, for example in community care, emergency departments and triage. Some of these changes had already been identified during the initial PReCePT pilot phase and been codified in the QI toolkit and implementation guide. For example, one of the first actions taken by implementers was to compare their hospital clinical guidelines with the PReCePT QI clinical guideline included in the toolkit. All units reported their unit to already have MgSO4 guidelines in place, but amendments were needed for these to reflect national policy and PReCePT QI protocols. Changing existing guidelines was the most often reported change implemented in units. Guideline updates included administration of MgSO4 for neuroprotection, addressing repeat doses, and most units had adjusted the gestational threshold of eligible pregnancies to include women up to 34 weeks, as described below:

We’ve changed, a hundred percent. We chose, in our unit, to offer [MgSO4] to everyone up to 33 weeks and 6 days because our numbers are so small […] so it’s become the norm really to give it. […] (P15, Midwife, ESP unit23)

Others structural impacts were ‘discovered’ and addressed during the implementation process, as the following interview excerpts illustrate:

You need an extra person (…) to go and do the magnesium sulphate because of all the other things that need to be done if somebody is in pre-term labour. Somebody else needs to go away to do it and we’ve appreciated that more I think. (P01, Midwife, ESP unit31).

What we had to do was to drill it into our registrars [doctors-in-training] that if somebody comes in, in preterm labour you don’t just write up magnesium and walk away, because then the poor midwife (…) she’s got to monitor the baby, she’s got to get a resuscitaire^4^ ready, she’s got to give her dexamethasone, she’s got to cannulate her, and then you expect her to give her the dose of magnesium as well, and then you wonder why when she delivers one hour later she’s not had the magnesium. So we drilled it into our registrars that you have to cannulate [the woman], you just give them magnesium straight away, you do it. (P27, Obstetrician, SSP unit15)

Workflows were actively restructured to remove barriers to women receiving MgSO4 in preterm labour, but they also restructured staff’s prioritising and decision-making. This is highlighted in the following excerpt describing the introduction of ‘PReCePT QI grab boxes’, resembling the ‘steroid boxes’ already in use. This was a box containing all the equipment and documentation needed when administering MgSO4. It was easily accessible in all places where MgSO4 needed to be administered such as in labour wards and operating theatres. These boxes helped make MgSO4 visible and act as a reminder, making administration as easy and quick as possible:

The biggest changes were essentially people’s mind-set, the thinking, just whenever somebody thinks of preterm labour, they not only have to think of transferring the baby, […] so in-utero transfers, steroids and magnesium sulphate. So we kept what we called grab box, so magnesium sulphate is available. This particular client comes through the door, we can just get hold of the whole bag, it’s all ready to go. (P43, Obstetrician, ESP unit23)

The ‘grab box’ was not only of practical value in reducing delays but also signalled the unit’s commitment to neonatal safety. Other normative restructuring changes included modifications to the way information was documented in patient notes: mainly clinical proformas and stickers to facilitate better recording of MgSO4 administration in maternity notes and easier transfer of information from maternity to neonatal databases. A powerful tool to improve uptake were the reviews of missed cases where audits revealed that women who should have received MgSO4 but did not, with findings being fed back into the system (through training, communication of results during meetings and handovers and one-to-one discussions). All units, irrespective of the level of implementation support they received, achieved some or most of the normative restructuring needed to increase their unit’s MgSO4 administration rate through use of the QI toolkit, implementation guide and ongoing QI support. This partly explains why there were no significant differences in MgSO4 administration rates between the ESP and SSP unit in the clinical trial results.

### Relational restructuring

Relational restructuring refers to how professional relationships and communication between different hospital units changed as a result of working with PReCePT to implement the new practice. Professional silo working was one of the greatest challenges for implementers who needed to promote perinatal team working, as the following excerpt highlights:

Obstetricians and neonatologists sometimes have a default tendency to operate in silos (…) the most optimistic interpretation I think I can give you is that the perinatal team is starting to form. (AHSN Clinical Lead 1)

Participants’ accounts suggest that poor team working was especially risky for the structural reasons explained above: vital information was not shared because it was stored in different locations, and communication was therefore suboptimal. The geographical distance between some maternity and neonatal units frequently exacerbated communication problems. Yet the care of women in preterm labour and the timely administration of MgSO4 required new routines that needed to be aligned with established responsibilities and a vision for joint working across maternity and neonatal unit staff. PReCePT activities (such as joint workshops and meetings, awareness training in different settings) enabled the perinatal team to engage in conversations about MgSO4 away from pressurised clinical environments, to develop networks across units and to raise and discuss concerns. They also enabled midwives to initiate conversations with obstetricians about when the administration of MgSO4 would be appropriate. These conversations facilitated a coming together of the perinatal team and the opening of opportunities for developing creative solutions to structural problems and for learning and improving practice, as illustrated below:

I think it [PReCePT] did have an impact as a joint project that everybody was involved with as a whole unit [….]. It gave the neonatal team the permission to say “is the mag sulph going up?” It gave the midwifery team permission to say “shall we start mag sulph?” And I think it was good that everybody was trying to do the same thing. (P01, Midwife, ESP, Unit31)

These outcomes were achieved more easily in ESP units that benefitted from additional backfill time for implementers drawn from all three professions (midwifery, obstetrics and neonatology) as well as additional events and meetings, focused training and coaching in QI methodology. Overall, there was more engagement from all three professions in the ESP units, while in the SSP units most of the implementation activities were carried out by midwives, as the excerpt below indicates:

It’s good having a midwife with dedicated time to go around doing some teaching […] To be honest a lot of that stuff I was doing I was juggling with other stuff, so I wasn’t doing it very well. […] At least now we’ve got a midwife there and she’s on labour ward all the time, whereas I’m all over the place. (P27, Obstetrician, SSP15)

However, one of the most notable differences between the two types of support was the way that PReCePT was viewed; in the ESP units, it was understood as a perinatal team project, involving all professions equally, whereas SSP units relied heavily on the lead midwives to support what was seen as either an obstetric or neonatal project. There was also less active involvement of obstetricians and neonatologists in SSP units. The strength and quality of these horizontal relationships had implications for the implementation process in ESP and SSP units. ESP units focused on collaboration, commitment and shared learning among participating units and invested in opportunities for this to happen. Regional and national support networks with which implementers engaged throughout the life of the study helped form ‘communities of practice’ within which knowledge was created and shared [21] and helped increase MgSO4 uptake in individual units, further enabling spread and scale-up of PReCePT QI. Another corollary of the enhanced QI support was the creation of ‘networks of networks’ where those participating in the PReCePT network also acted as links and access points to other networks, such as quality and safety collaboratives and local learning systems. This generated synergies which allowed the PReCePT message to be embedded within the wider system, raising the profile of MgSO4 as an important aspect of neonatal safety and transcending professional boundaries of responsibility.

These networks were facilitated by the national PReCePT team for ESP units but were lacking in SSP units resulting in fewer opportunities for implementers to be part of these mutually supportive, interprofessional collaborations. Nevertheless, clinicians and AHSN staff with QI and coordination roles also organised opportunities for local and regional meetings for training and exchanging knowledge and learning, replicating some but not all of the functions of the collaborative support received by ESP units. The AHSN support tended to focus on the SSP unit lead midwives who were seen as the main implementers. The SSP unit lead midwives were also highly proactive and creative in connecting with each other and seeking solutions to commonly experienced barriers and problems. For example, early in the implementation period, they formed a social media group as a peer support and information sharing tool and as a mechanism for spreading improvement ideas developed in local units. Inevitably, there was contact between ESP and SSP units, and while it may have been preferable to avoid cross-study arm contacts for the clinical trial to test the effectiveness of the enhanced intervention, in reality, this was impossible. Wider support networks encouraged commitment, motivation, exchanging ideas and networking which increased awareness and spread of MgSO4 administration.

Overall, relational restructuring was more challenging for SSP units. Midwives reported that it was often difficult to protect their time from clinical pressures, despite the funded backfill. This also had an impact on their capacity to attend training and meetings and complete tasks related to PReCePT such as training and awareness raising among staff in their units and hospitals, accurate data collection, data auditing and the investigation of missed doses and follow-up actions. These tasks were often carried out in their own time. Although ESP unit midwives were also called on to provide clinical support during their dedicated ‘PReCePT time’, they were better supported by their fellow implementers, and momentum was less likely to be lost during times of high pressure.

One way of securing more support for their efforts involved SSP unit midwives forging alliances with other members of the multidisciplinary obstetric and neonatal teams. For example, advanced neonatal nurse practitioners were enthusiastic supporters of MgSO4 uptake as the following excerpt illustrates:

It’s now become a sort of midwife-advance neonatal nurse practitioner led project […]. It’s become us two sort of leading it […] Our plans are to carry on the monthly meetings even once the PReCePT support has finished so we can maintain that. (P14, Midwife, SSP unit36)

Vertical relationships with the senior hospital leadership were not explicitly restructured as a result of implementation. However, they formed an important part of the context because explicit leadership support meant that structural and practical barriers with bureaucratic systems and policies could be overcome. Where that support was missing, implementers had difficulties in accessing PReCePT funds, and some hospital policies prevented the use of some parts of the PReCePT toolkit. However, this was not related to whether units received the SSP or ESP.

### Sustainment

Sustainment refers to how changes have become incorporated into routine practice following the ending of the implementation period, QI support and backfill funding. This coincided with the beginning of the COVID-19 pandemic. Sustainment of the increases in the administration of MgSO4 required ongoing work including the continued (re)evaluation of performance, dissemination and review of audit results, the identification of ‘missed cases’ and subsequent action to promote MgSO4 administration and address barriers. Improvements in data collection introduced in the implementation phase, regular training updates for existing staff and incorporating PReCePT training into mandatory staff induction programmes continued into the post-implementation phase, although some participant accounts suggested that clinical pressures and the impact of the COVID-19 pandemic led to falls in their administration rates. Reasons included staff shortages and reliance on untrained and agency staff, while opportunities for training were also reduced. Reported threats to sustainment included staff turnover and the resultant loss of QI expertise, the loss of protected midwife time through dedicated funding and potential competing demands from other safety initiatives.

Quantitative evaluation data [11] suggested that ESP units were more likely to sustain their administration rates, and that this was related to stronger perinatal team working. Team working had been encouraged in ESP units through implementer backfill time, the inclusion of all three professional groups responsible for maternal and neonatal safety in the implementation and opportunities for joint learning and networking, resulting in relational restructuring which in turn was critical to sustainment. Participants’ themselves also observed the importance of team working for sustainment:

It was good to have time with your team to talk about things which you normally just don’t have. Normally you always do things in between, […] and now you had the time to talk so it definitely improved our communication within the team and the collaboration with the neonatal unit. (P39, Obstetrician, ESP, Unit31)

I think when we do PReCePT compared to what we were doing in the past, we succeeded to train everyone and everybody has to sing from the same hymn book. […] the thing is even if one or two people were not doing it, […] then these people are going to stand out like a sore thumb, and they will (need to change their practice) and that’s what we can do now. (P25, Neonatologist, ESP, Unit07)

Participants also suggested that sustainment was strengthened by the inclusion of a wider membership in the perinatal team such as obstetric anaesthetists, pharmacists and advanced neonatal practitioners. Furthermore, participants’ accounts from both ESP and SSP units indicated that enhancing QI capacity in the workforce also had positive impacts on teams and individuals and their motivation to improve MgSO4 uptake. Finally, the use of social media, linking individual implementers to peer support, created a national forum for what was growing into a national community of implementers and became a powerful driver for the sustainment.

## Discussion

The evaluations of the National PReCePT Programme and the embedded randomised controlled trial (the PReCePT QI study) showed that increases in the uptake of MgSO4 were achieved across all units in England irrespective of whether they received a standard or enhanced QI package [11, 12]. The qualitative process evaluations provided some explanation. This paper focuses on three of the NPT constructs relating to outcomes that provide insights into how increases in uptake were accomplished: normative restructuring, relational restructuring and sustainment. While the technical component of the clinical intervention was straightforward, it was the work of integrating the change into the wider ecology of clinical practice including the policy/regulatory context and the organisational, team and individual practitioner levels that required considerable thought and effort. The findings suggest that the restructuring work, especially in relation to perinatal team processes, was vital to enable adaptations to be made and collective action to be taken and sustained. This study has demonstrated that normative restructuring was more easily accomplished than relational restructuring because it required practical, easily identifiable and observable changes. This allowed all units irrespective of the intensity of the implementation support to make improvements in their administration rates. The work of negotiating structural changes in perinatal team working —the relational restructuring — when implementation involves the coordination of professional working in different specialities with responsibilities to different patients was more challenging. Relational complexities were high because the patient benefiting from the intervention (in this case the prevention of neurological damage in the infant following premature birth) is different from the patient receiving the intervention (the woman in premature labour). This highlights the importance of attending to the ‘soft periphery’ of implementation [22] as well as its technical ‘hard core’ [22], the element that carries the key benefit, in this case administering a drug to a women with diagnosed premature labour. The ‘soft periphery’, including the changes in structures and relationships involved in delivering the ‘hard core’, was highly complex. The multidisciplinary steering group carefully considered the ‘soft periphery’ and produced the comprehensive QI toolkit and implementation guide following piloting and refinement so that it could be used flexibly by implementers and tailored to conditions in their own unit [10]. The decision which components to use and which to drop had to be made by local implementers depending on their units’ unique contexts. The piloting in a small number of maternity units, and integrating and codifying the learning, had been a crucial stage in the process [23]. The resultant QI toolkit and guide were available to all units. Similarly, all units received QI support although in different ‘doses’ through the SSP and ESP. As Dixon-Woods suggests [24], QI without targeted contextual support is likely to have limited impact.

The emergence of professional coalitions through the PReCePT programme at local, regional and national levels also supported relational restructuring. Clinicians worked as advocates for the improvements, legitimised changes, provided training and contributed their expertise to secure commitment and drive motivation [7, 24]. Indeed, peer pressure through comparing performance via data dashboards was an important way of influencing peers’ behaviours to make improvements. While lead obstetricians and neonatologists worked predominantly through their peers, the lead midwives’ implementation role was more complex. They acted as boundary spanners, a bridge between professional silos [25], engaged in relationship building [26], taking on multiple roles, navigating boundaries and accelerating change [27]. They bore the burden of driving the normative restructuring, often at a high cost to themselves. They were more likely to have been supported in implementation activities by the other clinical leads if they worked in ESP units. The lack of difference in the programme outcomes across all units can therefore be said to be largely down to their efforts in bringing about the improvements and their regional AHSNs who enabled the formation of peer support and communities of practice.

### Strengths and limitations

The strength of this study is the insights it gives into reasons why increases in the uptake of MgSO4 occurred in all units, irrespective of the level of QI support they received. Given the inevitable social contacts and knowledge sharing between the ESP and SSP units during the evaluation period, this was not surprising. However, focusing on the implementation outcomes specified in NPT, we showed that all units were successful in the normative restructuring of their setting which suggests that this implementation outcome is necessary to achieve improvements. However, it may not be sufficient to sustain and normalise the changes once additional support and resources have been withdrawn. Sustainment, our findings suggest, requires the restructuring of relationships and behavioural norms [28] to accommodate altered workflows and facilitate the sharing of responsibilities and tasks in daily working practices. The relational restructuring was more likely to have been achieved by ESP units but also happened in SSP units especially in those where perinatal team working was already established. The study has offered theoretical insights into restructuring processes that so far have not been well understood [7] based on empirical evidence.

The findings are limited insofar as data were only collected from individuals in implementation roles. The views from other staff members were not included. Ethnographic observations would have also enhanced the richness of the data and may have yielded finer-grained interpretations. However, what our data may have lost in depth, they gained in breadth due to the number of units participating in the process evaluation, also adding to the development of NPT outcome constructs.

## Conclusion

Unlike other large QI-focused spread and scale programmes which failed to show any impact on outcomes [20, 29, 30], the PReCePT programme in both the enhanced and standard support packages led to improvements in the uptake of MgSO4. An important insight of the qualitative components of the evaluation was that QI programmes require careful development with input from all people affected by the changes, attending to the normative and relational restructuring required to bring about improvements and piloting and codification of learning through accessible and flexible materials. But however well materials may have been designed, local implementers needed to be supported to translate the changes into their own context, giving them the opportunity to experiment, discover and be creative with material, financial and team resources. Changes were much more likely to occur in settings where interprofessional relationships were already strong and where there was a history of improvements, participation in research and QI projects, openness and a commitment to high standards of clinical practice. The findings suggest that QI capacity building irrespective of formal, nationally driven programmes might be useful given that QI interventions and clinical contexts are co-constitutive and that QI programmes interacted with the enabling factors already present in the setting [31]. A standard package with minimal support was therefore sufficient in settings with these enabling factors, but enhanced support was required in units where these were absent.

## Data Availability

The datasets generated and/or analysed during the current study are not publicly available to protect the anonymity of staff and their employers but are available from the corresponding author on reasonable request.

## Abbreviations

ESP: Enhanced support package
MgSO4: Magnesium sulphate
NPP: National PReCePT Programme
NPT: Normalisation process theory
PReCePT: Prevention of Cerebral Palsy in PreTerm Labour
QI: Quality improvement
SSP: Standard support package

## Footnotes

1 There are 155 maternity units in England. The 5 units that were PReCePT pilot sites in the West of England were not included in the evaluations.

2 The analysis of these data preceded the publication of NPT coding manual.

3 Perinatal teams comprise midwives, obstetricians, neonatologists and neonatal nurses.

4 A resuscitaire is a piece of equipment for the resuscitation and warming of new-born babies.
